# Transition Metal Ion Complexation and Extraction by Hydroxamate Functionalised p-Tert-Butylcalix[4]Arenes

**DOI:** 10.1155/MBD.1994.151

**Published:** 1994

**Authors:** Jeremy D. Glennon, Elizabeth Horne, Patricia O'Sullivan, Sharon Hutchinson, M. Anthony McKervey, Stephen J. Harris

**Affiliations:** 1 Chemistry Department University College Cork Ireland; 2 School of Chemistry The Queen's University of Belfast Ireland; 3 Dublin City University Ireland

## Abstract

The functionalisation of macrocyclic p-tertbutylcalix[4]arenes at the lower rim with hydroxamic acid and proline hydroxamic acid groups gives the calixarenes chelating properties similar to siderophores.  The ^1^H NMR spectrum of p-tertbutylcalix[4]arene tetrahydroxamic acid shows a broad band at 10.8 ppm in DMSO-d_6_ attributed to NHOH protons.  Diffuse reflectance spectral analysis of PVC membranes containing the calixarene hydroxamic acids show absorption bands at 485 nm and 504 nm following contact with aqueous solutions of Fe(III) and V(V) respectively. The pH dependency of this extractive selectivity is examined for Fe(III), Co(II), Pb(II), Mn(II), Cu(II) and Ni(II) using a solid phase extraction approach.   Spectrophotometric evidence for the complexation of Cu(II) in methanol by p-tertbutylcalix[4]arene tetraproline acid is also provided.


### TRANSITION METAL ION COMPLEXATION AND EXTRACTION BY HYDROXAMATE FUNCTIONALISED P-TERT-BUTYLCALIX[4]ARENES

Jeremy D. Glennon*[1], Elizabeth Horne[1], Patricia O'Sullivan[1], Sharon Hutchinson[1], M. Anthony McKervey[2] and Stephen J. Harris[3]

[1] Chemistry Department, University College Cork, Ireland
[2] School of Chemistry, The Queen's University of Belfast, N. Ireland
[3] Dublin City University, Ireland

### Abstract

The functionalisation of macrocyclic p-tertbutylcalix[4]arenes at the lower rim with hydroxamic acid and proline hydroxamic acid groups gives the calixarenes chelating properties similar to siderophores. The $^{1}H$ NMR spectrum of p-tertbutylcalix[4]arene tetrahydroxamic acid shows a broad band at 10.8 ppm in DMSO-$d_6$ attributed to NHOH protons. Diffuse reflectance spectral analysis of PVC membranes containing the calixarene hydroxamic acids show absorption bands at 485 nm and 504 nm following contact with aqueous solutions of Fe(III) and V(V) respectively. The pH dependency of this extractive selectivity is examined for Fe(III), Co(II), Pb(II), Mn(II), Cu(II) and Ni(II) using a solid phase extraction approach. Spectrophotometric evidence for the complexation of Cu(II) in methanol by p-tertbutylcalix[4]arene tetraproline acid is also provided.

### Introduction

Hydroxamate siderophores are of biomedical importance because of their uses in chelation therapy, in particular for the removal of Fe(III) and Al(III) from the body. Siderophores are produced by selected microorganisms when grown in culture media under iron-limiting conditions and act as sequestering agents for Fe(III) incorporation into the microorganisms[1]. These sequestering agents have in general high stability constants for the complexation of Fe(III), due mainly to the presence of hydroxamic acid or catecholate functional groups. The chemical structure of the important chelation therapy drug, DFA, used in the treatment of iron overload, is given in Figure 1.

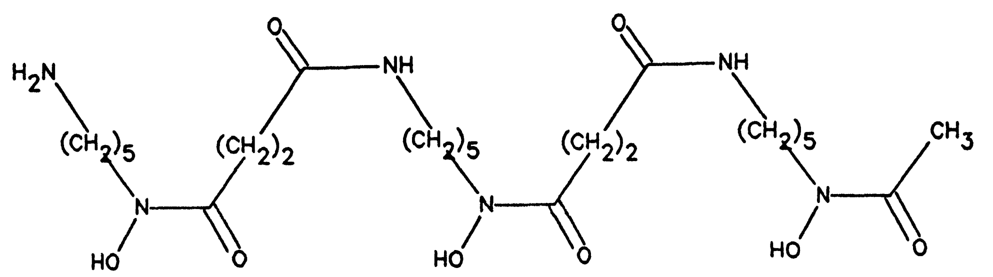

Figure 1: Chemical Structure of the trihydroxamic acid, Desferrioxamine (DFA).

Chelation therapy using Al(III) complexation is also important because of the association of Al with a variety of neurological dysfunctions and bone disorders[2,3]. A related biomedical example is dialysis dementia, where accumulation of Al(III) occurs during renal dialysis, an effect that can be avoided by the prior removal of this metal ion from the water supply through extractive complexation. As a result of these applications of chelation, there is now a renewed interest in designing new ligands for the complexation of these important metals with a view to obtaining more effective and selective chelation[4].

In particular, synthetic macrocyclic compounds with designed intramolecular cavities are providing greater steric control over the optimisation of selectivity of metal binding. One such example of ion recognition, is provided by calixarene compounds which are the macrocyclic products of phenol-formaldehyde condensations. When suitably functionalised at the lower rim, selective complexation of cations is produced. This selectivity is demonstrated through phase transfer studies of alkali metal cations from aqueous solution into dichloromethane[5], ion selective electrode applications[6,7] and chromatography[8]. Functionalisation of the calixarenes with suitable ligands produces chelating macrocyclic compounds for the complexation of such metal ions as Fe(III) and Cu(II). Specifically, the incorporation of hydroxamic acid groups on p-tert-butylcalix[6]arene has been shown to produce high extractive selectivity for $UO_2^{2+}$ ions[9]. In this work, tetrameric calixarene hydroxamic acids (Figure 2) are examined as complexing agents for transition metal ions such as Fe(III), Co(II), Pb(II), Mn(II), Cu(II) and Ni(II). The pH dependency of metal ion extraction is examined and spectroscopic evidence for calix[4]arene hydroxamic acid complexation of Fe(III) and V(V) is provided.

### Materials and Methods

### Materials

Unless otherwise stated, all chemicals were analytical reagent grade quality. Ethylenediaminetetraacetic acid disodium salt (EDTA) was purchased from BDH (Poole, England) while the octadecylsilica, LiChroprep RP-18 (25-40 $\mu$m) was obtained from Merck. The membrane components, PVC (ISE grade) and Analar grade tetrahydrofuran (THF) were obtained from Fluka. Dioctyl phenyl phosphonate (DOPP) (plasticiser) was obtained from Aldrich. Analar grade salts, ferric nitrate and ammonium metavanadate were obtained from BDH, (Poole, England). Analar grade nitric Acid and sulphuric acid were obtained from Riedel de Haën.

Metal ion stock solutions of 100 ppm were made up from their nitrate salts (Merck Titrisol 1000 ppm standards) for the solid phase extraction studies. Nitric acid (conc.), potassium hydroxide (5M) and 35% ammonia solution were used for pH adjustments. Deionised water from an Elgastat spectrum water purification unit, with a resistivity of 15 M ohm $cm^{-1}$ or better was used. All glassware was routinely acid washed. The calixarene hydroxamate, 25,26,27,28-tetrakis(N-hydroxycarbomoylmethoxy)-p-tert-butylcalix[4]-arene was supplied by Loctite (Ireland) Ltd. and synthesised as previously described[10] (Fig. 2). The p-tertbutylcalix[4]arene tetra-D,L-proline hydroxamate and the tetra-L-proline acid were synthesised at UCC, Ireland by McKervey et al as previously described[11].

### Methods

### Membrane Preparation for Diffuse Reflectance Analysis

The membrane components (0.6% m/m calixarene hydroxamic acid, 70.2% DOPP, 29.2% PVC) were mixed and dissolved by stirring overnight in THF. The solution was poured into a glass mould and on slow evaporation of the THF, a clear flexible membrane was formed. The membrane was soaked in 10 mls 0.05 M Fe(III) solution or V(V) solution for 30 mins, removed, rinsed with water and air dried. Diffuse reflectance spectra were recorded for both membranes and for a membrane unexposed to the metal solutions.

### Metal Ion Extraction by calix[4]arene hydroxamates on ODS

A set amount of LiChroprep RP-18 (25-40 $\mu$m) (i.e. 0.35g) was added to a plastic container (1.25 x 0.9 cm i.d.) fitted with a frit at its base and methanol was pumped through to wet the octadecylsilica. Thereafter, a suspension consisting of 0.094g calixarene hydroxamate in 10 ml methanol was slowly passed through the octadecylsilica using a pressurised glass syringe. Another frit was placed on top of this silica bed and pressure was applied to ensure that the bed was tightly packed. The cartridge was connected to the peristaltic pump and water was pumped through at a rate of 1 ml $min^{-1}$.

Metal solutions (5 ppm, 25 ml) were adjusted to the required pH and pumped at a rate of 1 ml $min^{-1}$ through separate cartridges containing calixarene hydroxamates 1 and 2 (Fig. 2). This was followed by 25 ml of water to wash off any remaining uncomplexed metal ions. Thereafter the metal was eluted with 25 ml of 0.01 M EDTA or acidified water (pH 2.0). Subsequent flame atomic absorption spectrometric or ion chromatographic analysis gave the % uptake of the various metal ions and % recovery of the eluted metals.

### Visible Absorbance Spectra of Cu(II)-p-tertbutylcalix[4]arene tetraproline acid

NaOH (1.05 M) was added to a $2 \times 10^{-3}$ M solution of the calixarene in MeOH to quantitatively titrate the tetraproline carboxylic acid protons. Aliquots (10 $\mu$l) of a copper nitrate solution (0.15 M in MeOH) were added to 3 ml of the calixarene solution to give Cu(II): calixarene mole ratios in the range 0.25 - 3.0 and the uv-visible absorption spectra recorded.

Absorbance values taken at 661 nm and 725 nm were plotted against the mole ratio values.

### Instrumentation

$^{1}H$ NMR Spectra were recorded in DMSO-$d_6$ using a Jeol 270 MHz NMR spectrometer. Diffuse reflectance spectra were recorded using a Shimadzu UV-3101 PC UV-Vis-NIR Scanning Spectrophotometer. A cartridge (plastic container, 1.25 x 0.9 cm i.d.) containing the calixarene hydroxamate on ODS was used in association with a masterflex peristaltic pump (Cole-Parmer Instruments, Chicago, IL., USA). An Orion research model 231 pH meter was employed for the pH measurement of all metal ion solutions. Ultraviolet and visible absorption spectra were recorded on a Cary IE UV-visible spectrophotometer. Atomic absorption analyses were carried out using a Pye Unicam SP9 spectrophotometer. Ion chromatographic analysis was carried out using a Dionex 4500i ion chromatographic system with a CS5 cation separator column.

### Results and Discussion

Macrocyclic compounds such as the calixarenes under study here can be functionalised with polydentate groups, to yield selective metal ion receptor molecules. The selectivity of the resulting chelating calixarene is determined by the cavity size produced and the nature of the chosen ligands[4]. This selectivity, accompanied by a high stability constant, is often demonstrated by selective solvent extraction of the coordinated metal ion. Illustrative examples of this include the phase transfer of alkali metal cations as metal picrates from aqueous solution by calixarene esters in dichloromethane and the solvent extraction of Cu(II) from ammonia solution using p-t-butylcalix[6]arene[12].

### Metal Ion Complexation by hydroxamate functionalised p-tertbutylcalix[4]arenes

The chemical structures of the chelating calixarenes under study here for their complexation ability, in particular for Fe(III) and Cu(II), are given in Figure 2. These

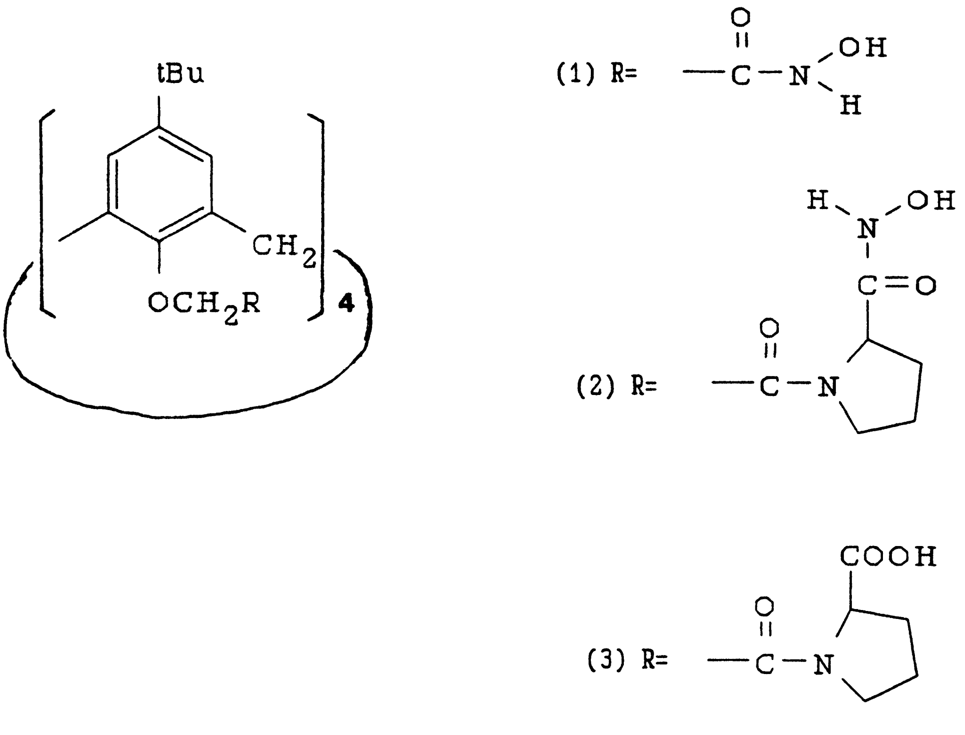

Figure 2: Chemical structures of p-tertbutylcalix[4]arene derivatives (1) tetrahydroxamic acid, (2) tetraproline hydroxamic acid and (3) tetraproline acid.

tetrameric calixarenes are functionalised at the lower rim to contain hydroxamic acid groups and are as such synthetic macrocyclic models of naturally occurring siderophores. The presence of the hydroxamic acid group on the calixarene is evidenced from the $^1H$ NMR spectrum of calixarene 1 in DMSO-$d_6$ (Figure 3). Characteristic doublets are evident for the methylene hydrogens of the calixarene backbone while a broad peak in the 8-12 ppm region can be attributed to the NHOH protons. The solid state IR spectrum shows an intense broad band $\nu(C=O)_{st}$ at ~ 1665 $cm^{-1}$ a characteristic feature of hydroxamate carbonyl groups[13].

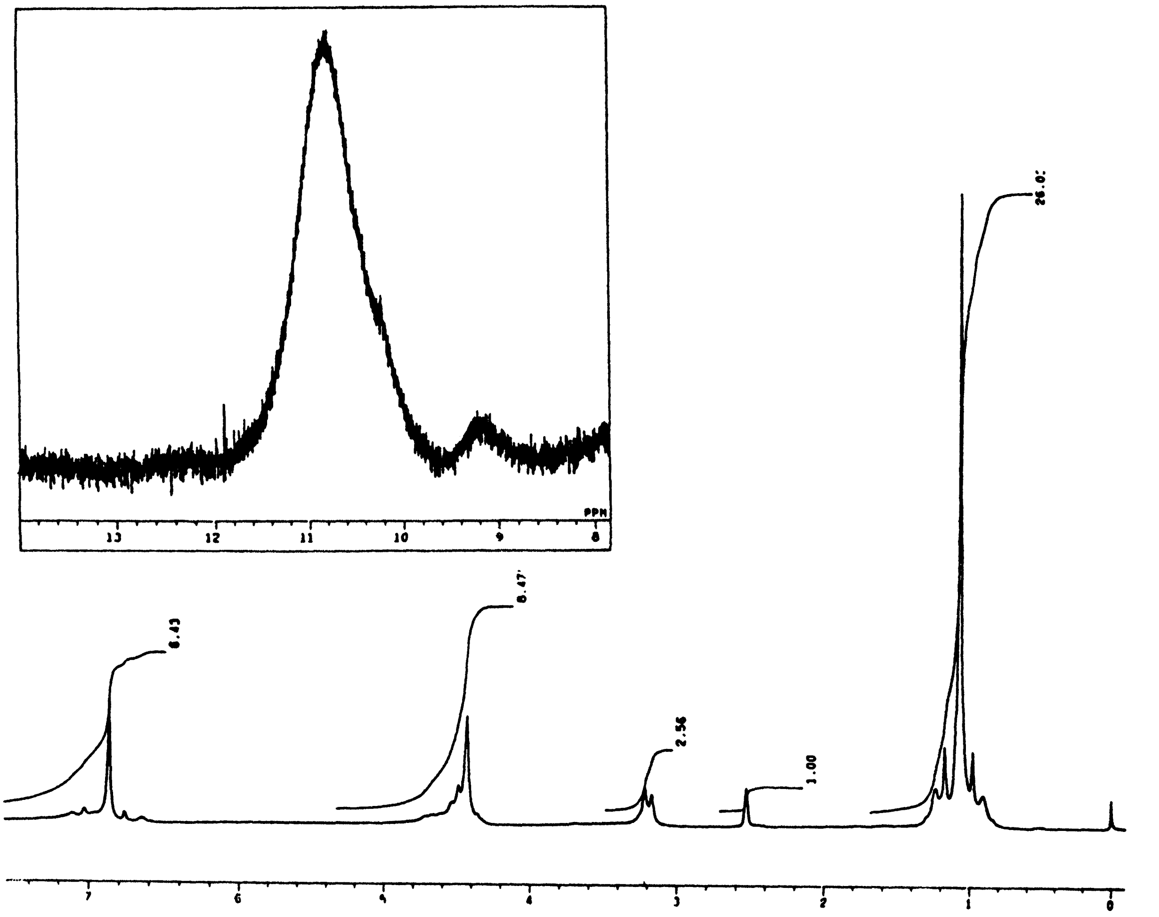

Figure 3: $^1H$ NMR spectrum of p-tert-butylcalix[4]arene tetrahydroxamic acid in DMSO-$d_6$.

Spectroscopic evidence for the extractive complexation of Fe(III) and V(V) by p-tertbutylcalix[4]arene tetrahydroxamic acid was gained from diffuse reflectance spectra. Thin PVC membranes containing the calixarene when placed in aqueous solutions of Fe(III) or V(V), developed red-brown colours with maximum absorbances at 435 nm and 504 nm respectively, as shown in Figure 4. The nature and extent of complexation by chelating calixarene hydroxamates to metal ions is determined by the degree of solvent receptor contact, kinetics and pH. Only limited information on the effect of pH on the complexation of calixarenes of this type is available, with many liquid-liquid extraction

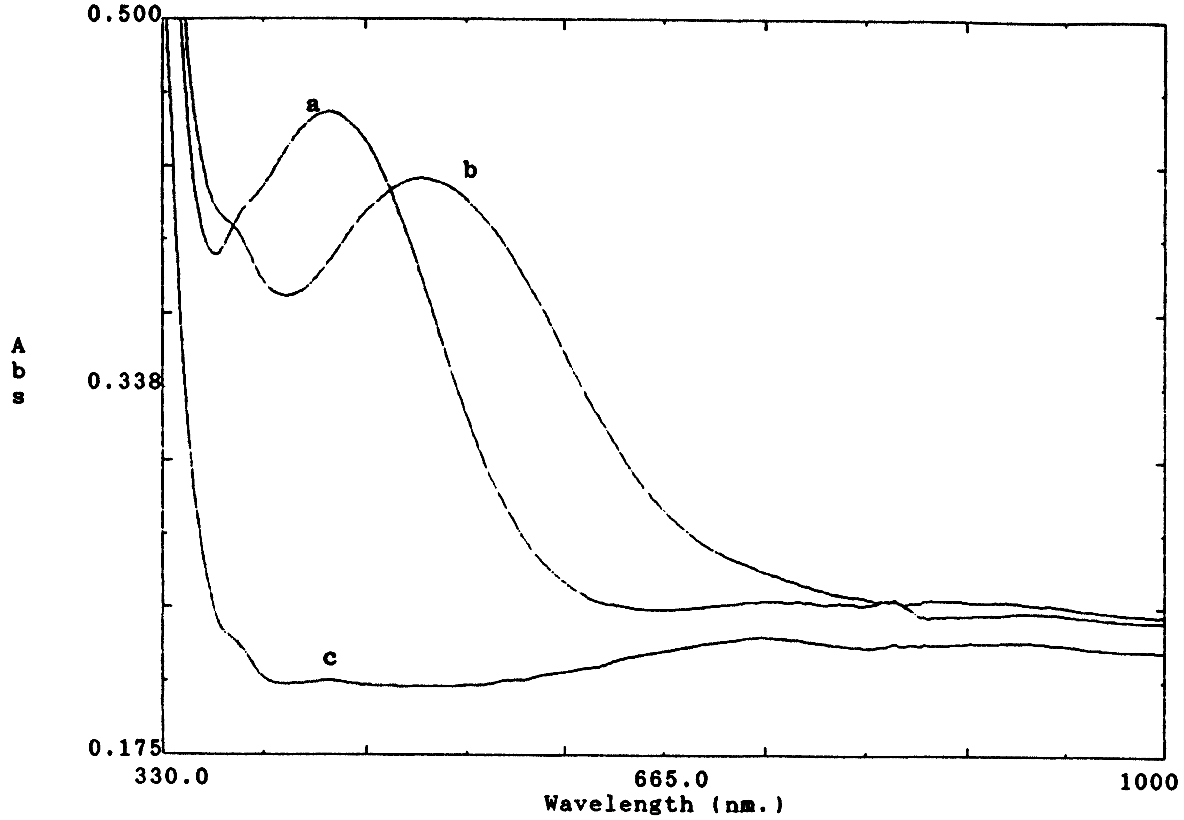

Figure 4: Diffuse reflectance spectra of Fe(III) and V(V) complexes of p-tertbutylcalix[4]arene tetrahydroxamic acid, in PVC membranes (a) Fe(III), (b) V(V), (c) metal free membrane.

studies being carried out at a fixed pH. By supporting the calixarenes on a solid phase sorbent such as octadecylsilica, and passing through aqueous metal ion solutions of varying pH, the full pH dependency of metal ion complexation and extraction is obtained. A typical metal uptake profile obtained for calixarene 2, the tetraproline hydroxamate is shown as a function of pH in Figure 5. With the exception of Pb(II), the pH dependency of the metal uptake curves is similar to those obtained by liquid-liquid extraction for N-

phenylacylhydroxamic acids in chloroform[14]. Cu(II) and Fe(III) are quantitatively extracted by pH 5 while Ni(II), Co(II), Zn(II), Mn(II) and Cd(II) require an alkaline pH for complete complexation. Pb(II) however is completely complexed by pH 3, whereas a pH of 7 is needed in liquid-liquid extraction of this metal ion using N-phenylacylhydroxamic acids. The p-tertbutylcalix[4]arenes functionalised with amide groups are known to be more extractive for the heavy metal cations such as $Pb^{2+}$ than ester containing derivatives. The low pH complexation of $Pb^{2+}$ was observed here for calixarene 1 and 2, suggesting that this behaviour is associated with the organised presence of hydroxamic acid functional groups on the lower rim of the calix[4]arenes.

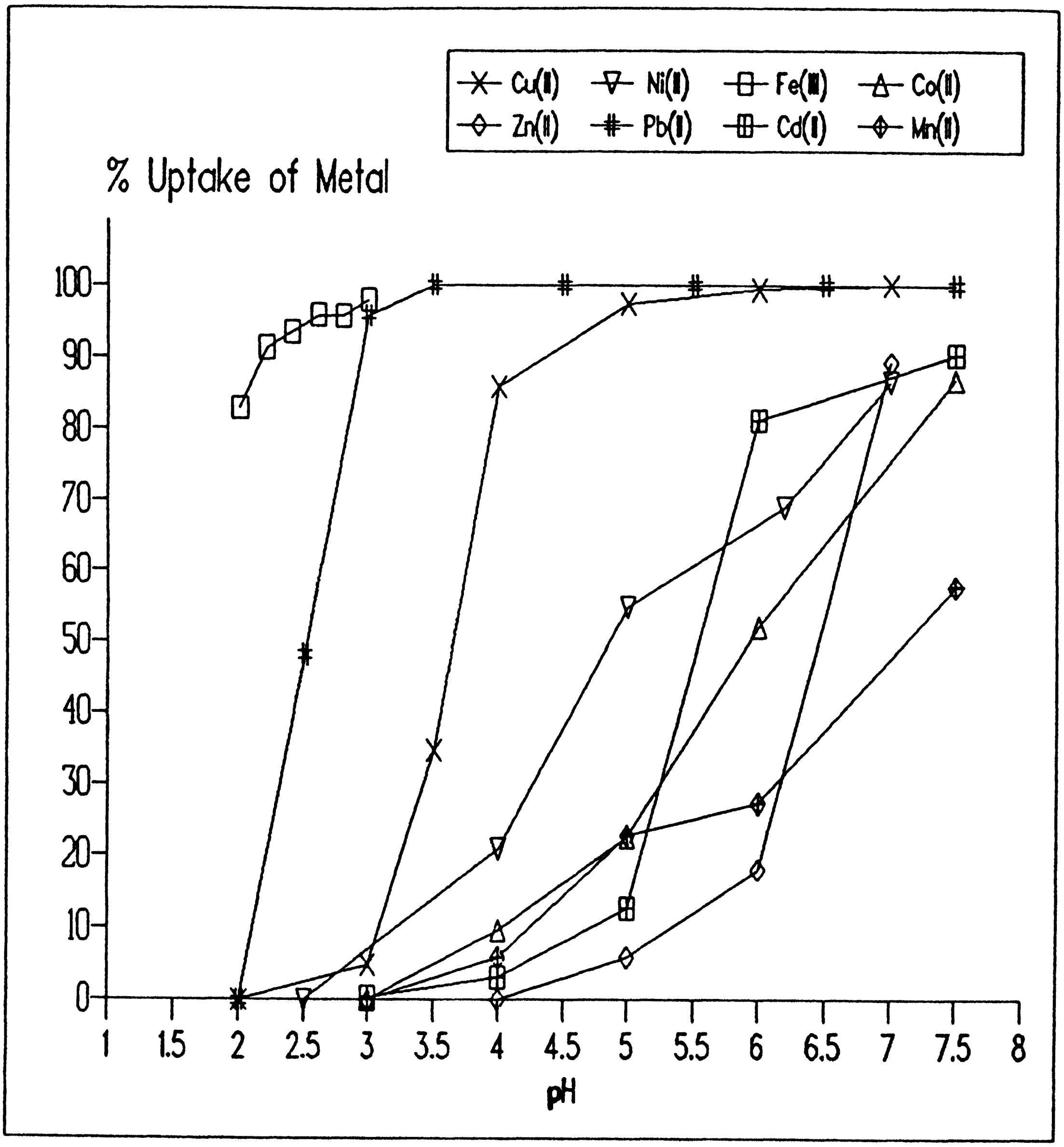

Figure 5: Effect of pH on metal ion complexation by p-tertbutylcalix[4]arene tetraproline hydroxamate supported on ODS.

**Cu(II) complexation to p-tertbutylcalix[4]arene tetraproline acid**

Solution studies in MeOH were carried out on calixarene 3 in the presence of Cu(II) ions using visible spectro-photometry. The direct addition of Cu(II) nitrate to the tetraproline acid in MeOH did not give rise to complexation of Cu(II). However the prior removal of protons from the four carboxylic acid groups by the addition of NaOH, resulted in the appearance of a broad absorption band between 500 and 750 nm on the addition of Cu(II). The spectra recorded following increasing addition of Cu(II) up to a Cu:calixarene ratio of 3.0 are shown in Figure 6. The wavelength maximum of 628 nm at a ratio of 0.25 moves to 661 nm at a ratio of 1.0 and shifts to above 700 nm at ratios higher than 2.0. Mole ratio plots of the absorbance changes at 661 nm and 725 nm show a well defined change in slope at the 1.0 ratio value, indicating the formation of a 1:1 complex with a maximum absorption at 661 nm.

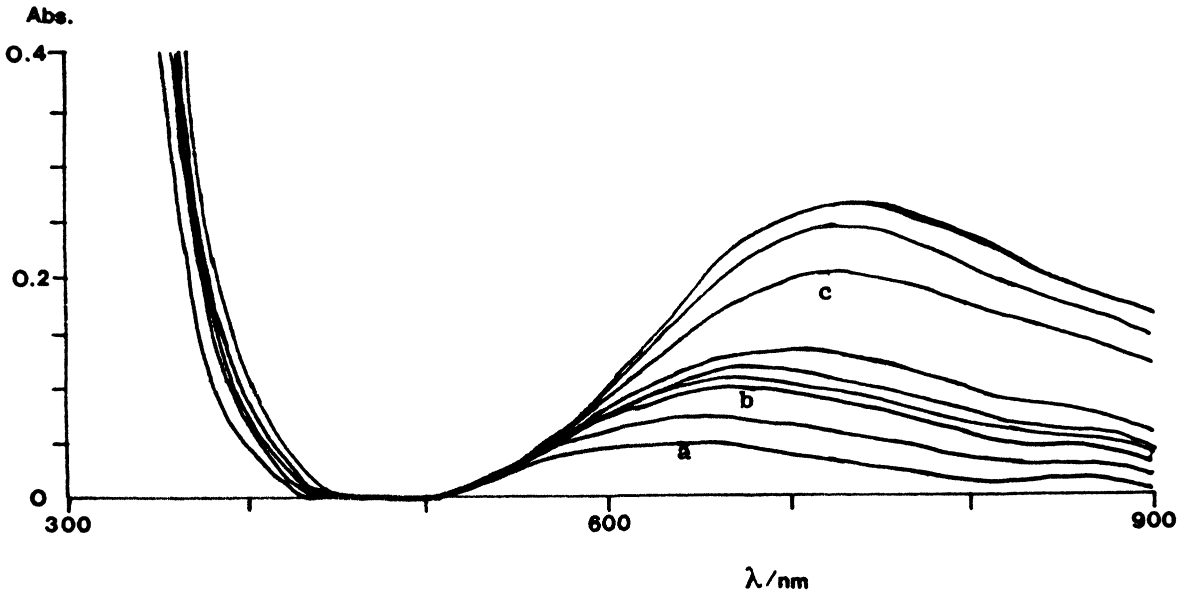

Figure 6: UV-visible absorption spectra of Cu(II): p-tertbutylcalix[4]arene tetraproline acid in MeOH. Metal ligand ratios (a) 0.5, (b) 1.0, (c) 2.0.

### References

1. Nielands, J.B., *Ann. Rev. Biochem.*, 1981, **50**, 715.
2. Kruck, T. et al, *J. Chromatogr.*, 1988 **433**, 207.
3. Clevette, D.J. and Orvig, C., *Polyhedron*, 1990, **9**, 151.

4. Evers, A., Hancock, R.D., Martell, A.E. and Motekaitis, R.J., *Inorg. Chem.*, 1989, **28**, 2189.

5. Arnaud-Neu, F. et al., *J. Am. Chem. Soc.*, 1989, **111**, 8681.

6. Cunningham, K., Svehla, G., Harris, S.J. and McKervey, M.A., *Analyst*, 1993, **118 (4)**, 341.

7. Odashima, K., Yagi, K., Tohda, K. and Umezawa, Y., *Anal. Chem.*, 1993, **65**, 1074.

8. Glennon, J.D., O'Connor, K., Srijaranai, S., Manley, K., Harris, S.J., and McKervey, M.A., *Analytical Letters*, 1993, **26 (1)**, 153.

9. Nagasaki, T. and Shinkai, S., *J. Chem. Soc. Perkin Trans. II*, 1991, 1063.

10. Harris, S.J., MacManus, M.G. and Guthrie, J., *European Patent E.P.* 0309291 A1 published March 29th 1989, Assigned to Loctite (Ireland) Ltd.

11. Cremin, S., Ph.D. thesis National University of Ireland 1991.

12. Yoshida, I., Fujii, S., Ueno, K., Shinkai, S. and Matsuda, T., *Chemistry Letters*, 1989, 1535.

13. Brown, D.A., Geraty, R., Glennon, J.D. and NiChoileain, N., *Inorg. Chem.*, 1986, **25**, 3792.

14. Hojjatie, M., Cecconie, T. and Freiser, H., *Analytica Chimica Acta*, 1987, **199**, 49-57.

**Received: September 2, 1993**

